# Switching of magnetic easy-axis using crystal orientation for large perpendicular coercivity in CoFe_2_O_4_ thin film

**DOI:** 10.1038/srep30074

**Published:** 2016-07-20

**Authors:** Sagar E. Shirsath, Xiaoxi Liu, Yukiko Yasukawa, Sean Li, Akimitsu Morisako

**Affiliations:** 1 Spin Device Technology Center, Faculty of Engineering, Shinshu University, Nagano 380-8553, Japan; 2 School of Materials Science and Engineering, University of New South Wales, Sydney, NSW 2502, Australia

## Abstract

Perpendicular magnetization and precise control over the magnetic easy axis in magnetic thin film is necessary for a variety of applications, particularly in magnetic recording media. A strong (111) orientation is successfully achieved in the CoFe_2_O_4_ (CFO) thin film at relatively low substrate temperature of 100 °C, whereas the (311)-preferred randomly oriented CFO is prepared at room temperature by the DC magnetron sputtering technique. The oxygen-deficient porous CFO film after post-annealing gives rise to compressive strain perpendicular to the film surface, which induces large perpendicular coercivity. We observe the coercivity of 11.3 kOe in the 40-nm CFO thin film, which is the highest perpendicular coercivity ever achieved on an amorphous SiO_2_/Si substrate. The present approach can guide the systematic tuning of the magnetic easy axis and coercivity in the desired direction with respect to crystal orientation in the nanoscale regime. Importantly, this can be achieved on virtually any type of substrate.

The pursuit of a fundamental understanding and the control of nanomagnetism have received significant interest due to its novel properties and wide ranges of potential applications. Examples of nanomagnetic materials include nanosized room-temperature ferromagnets for memory applications or materials with high magnetization and switchability that lend themselves to thin film architectures for device development. The ability to control magnetic properties such as saturation magnetization, remanent magnetization, and coercivity is important not only for a fundamental understanding of magnetism but also for the applications of magnetic data storage, MRI contrast-enhancement agents, and magnetic hyperthermia for biomedical therapeutic purposes^1^,^2^,^3^,^4^. Cobalt ferrite, CoFe_2_O_4_ (CFO), a ferrimagnetic inverse spinel, is one of the most commercially significant members of the magnetic ferrite class. CFO resides in an inverse spinel structure (space group ***Fd3m***; no. 227), which is a cubic crystal system with oxide anions arranged in a cubic close-packed lattice and metal cations tetragonally or octahedrally surrounded by oxygen (Fig. 1a). Each unit cell consists of 32 oxygen ions, 16 octahedral sites and 8 tetrahedral sites. For CFO, Fe^3+^ ions occupy the tetrahedral sites and half of the octahedral sites, whereas Co^2+^ ions are located at the remaining octahedral sites^5^.

Below the Curie temperature (Tc) of 860 K, CFO presents a long-range collinear ferrimagnetic order with antiferromagnetic intersublattice exchange interactions^6^. CFO has a magnetic easy axis along the (100) directions^7^,^8^ with a high saturation magnetization (400 emu/cm^3^), and it has a positive first-order magnetocrystalline anisotropy constant (***K***_***1***_ ∼ 2 × 10^6^ ergs/cm^3^), an order of magnitude greater than other spinel structure ferrites, resulting in a high coercivity (***Hc***). These properties make CFO as a unique material for magnetic spin filters^9^ and spintronics devices such as charge-/strain-driven multiferroic nanostructures^10^,^11^,^12^. It is a common material of choice used in magnetic recording media, and it serves as a ferromagnetic component of read/write heads^13^,^14^. All these applications depend upon the characteristic values of ***Hc*** and saturation magnetization (***Ms***) which is magnetic easy-axis dependent. Particularly in hard disk drives (HDD), the properties of magnetic recording media depend upon the relative orientation of the magnetic easy axis. A preferential perpendicular magnetization and anisotropy in CFO are necessary for a variety of application. Reports have shown a magnetic easy axis tailored through a lattice mismatch between film and substrate^7^,^15^,^16^, with very expensive single crystal or sapphire substrates. However, to obtain the perpendicular anisotropy, precise control over the direction of the magnetic easy axis of the CFO thin film deposited on an inexpensive amorphous substrate remains significant challenge; therefore, an effective strategic approach needs to be develop.

In this study, we demonstrate a fundamental approach for systematically modulating the maximized perpendicular magnetic anisotropy and magnetic easy axis of CFO thin film based on a consideration of the effects of crystal orientation, compressive strain anisotropy and oxygen vacancies. The (111)-preferred and oxygen-deficient (311) randomly oriented CFO thin films are fabricated by the DC sputtering technique and then systematically examined by various characterizations. We demonstrate that the oxygen-deficient film after post-annealing gives rise to a compressive strain perpendicular to the film surface. We extend this concept to develop a CFO thin film with large perpendicular coercivity in a desired direction with moderate saturation magnetization. Our strategy is given in a scheme where the *in*-plane and perpendicular coercivity are associated with a crystal orientation (Fig. 1). This approach can lead to the systematic tuning of the magnetic easy axis and coercivity, particularly with respect to crystal orientation in a nanoscale regime; importantly, this can be achieved on any types of substrate.

## Methods

### Thin film sample preparation

CoFe_2_O_4_ thin films 40 nm thick were grown on amorphous SiO_2_/Si substrate by DC sputtering. A commercially available ferrite with a stoichiometric composition of the CoFe_2_O_4_ target (Kojyundo Chemical laboratory Co., LTD) was used for the deposition of the CFO thin films. The chamber was evacuated to a pressure below 3 × 10^−4^ Pa, and then, the total working gas pressure of the Ar, 0.4 Pa, was fed into the chamber. The facing target sputtering (FTS) technique^17^ was used in the present work, which stoichiometrically transfers the composition from the target to the film, in which the distance between the plasma and the substrate was approximately 6 cm, and the DC power of 30 W was applied for the deposition. The samples were grown for 1 h (40 nm) with different substrate temperatures (Ts), viz. room temperature (RT), 50, 100, 150, 200, 250, 300, 325, 350 and 375 °C. After the deposition of film, the samples were subjected to post-annealing (Ta) at 400 and 800 °C.

### Characterizations

The prepared CoFe_2_O_4_ thin films were characterized by means of X-ray diffraction (XRD; Rigaku SmartLab). Pole figure analysis was conducted using ‘3D Explore’ software, reflection high-energy electron diffraction (RHEED; JEOL JEM-2010), X-ray photoelectron spectroscopy (XPS; AXIS-ULTRA DLD, Shimadzu), vibrating sample magnetometry (VSM; Tamagawa Factory Co. Ltd., custom-made), atomic/magnetic force microscopy (AFM/MFM; Veeco, Innova) and field-emission scanning electron microscopy (FE-SEM; Hitachi SU8000).

## Results and Discussion

**Figure 2:**
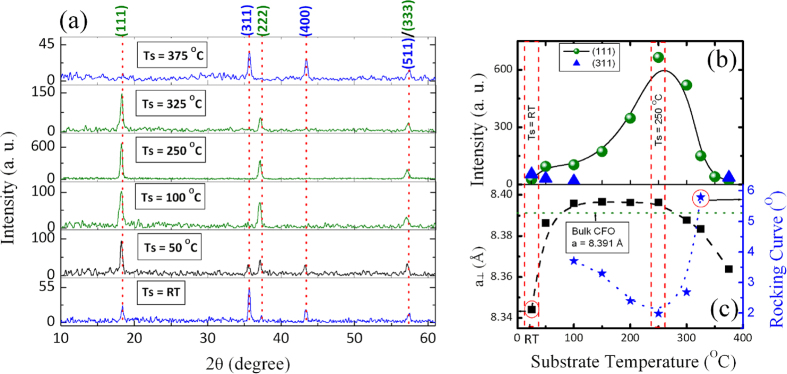
(**a**) X-ray diffraction θ–2θ scans (*background removed*) of post-annealed CFO. The XRD pattern of Ts = RT and Ts = 375 °C, blue in color, shows a randomly oriented CFO, XRD pattern of Ts = 100 to 325 °C; the green in color shows (111)-oriented CFO and XRD pattern; Ts = 50 shows the (111)-dominated random orientation of CFO thin film. The vertical dotted lines indicate the positions of the lattice plane reflections. (**b**) Variation of XRD peak intensity in the (111) and (311) planes with varying substrate temperatures. (**c**) Variation of the out-of-plane (a_⊥_, c-axis) lattice parameter and the rocking curve with the substrate temperature of the CFO thin film. The horizontal dotted line in part c indicates the lattice parameter of bulk CFO.

The X-ray diffraction patterns of 40-nm CFO thin film samples deposited at different substrate temperatures (Ts) are shown in Fig. 2(a). As seen in Fig. 2(a), the sample deposited at room temperature shows that the (311) diffract line is the most intense peak, indicating the random orientation of the CFO thin film. The XRD pattern shows a weak contribution from (111), (222), (400) and (333). It should be noted that no contribution from the (220) plane is detected in the randomly oriented CFO. As the Ts increased from 50 to 100 °C, the intensity of the (311) line decreased, and the (111) orientation became dominant with the group lines of (222 and 333). The polycrystalline CFO films generated XRD patterns with all possible crystal orientations of the material, which was similar to the bulk powders. However, (111) preferred oriented or textured films demonstrated XRD patterns with certain Bragg reflections that were more pronounced than others. It is of great significance to grow (111)-oriented CFO thin films on an amorphous SiO_2_/Si substrate at the low temperature of 100 °C. Most importantly, we observed that this (111) orientation was true for the as-deposited CFO film without any need for post-annealing. The further increase of Ts resulted in a similar pattern but with strong diffraction intensity. Figure 2(a,b) show that the CFO deposited at a Ts = 250 °C showed the strongest intensity for the (111) plane. The intense (111), (222) and (333) diffraction lines of the CFO spinel structure indicated a strong preferential orientation, with the (111) directions of the crystallites perpendicular to the film plane. As the Ts increased ≥ 350 °C, the (111) preferential orientation was not observed, and the CFO thin film again showed a random orientation. It is worth to mention here that, XRD peak at 2θ = 57.4 ° is corresponding to (333) and (511) planes and is observed in all the XRD patterns. The (333) plane is observed if the samples are (111) oriented and the plane (511) is observed when the sample is randomly oriented. The full width at half maximum (FWHM) of a rocking-curve analysis of the (111) plane of CFO thin film deposited at different substrate temperatures is shown in Fig. 2(c). CFO thin film deposited at Ts = 250 °C shows the lowest FWHM (1.9 °) of the rocking curve confirmed the high (111) orientation. The substrate temperature-dependent change in the crystal orientation of the investigated CFO thin film was related to the crystallographic change in the polycrystalline or epitaxial thin films, which was driven by a reduction in the system total anisotropy energy, i.e., E_total_ = [(***γ***_***s***_ + ***γ***_***i***_)/***h***] + ***M***_***hkl***_***ε***^***2***^, where ***γ***_***s***_ is surface energy, ***γ***_***i***_ is interfacial energy, and ***h***is film thickness^18^. The last term, ***M***_***hkl***_**ε**^**2**^, is elastic strain energy density, ***ε*** is intrinsic residual strain, and ***M***_***hkl***_ is the biaxial modulus, which is closely related to the crystallographic direction. It should be noted that we used an amorphous SiO_2_/Si substrate, and therefore, ***γ***_***i***_ can be neglected.

The ***γ***_***s***_ for different planes of CFO are anisotropic and are: ***γ***_***s***(***111***_) (208 ergs/cm^2^) < ***γ***_***s***(***400***_) (1486 ergs/cm^2^) < ***γ***_***s***(***220***_) (1916 ergs/cm^2^) *<* ***γ***_***s***(***311***_) (2344 ergs/cm^2^)^19^. The closed-packed (111) planes of the CFO have the lowest surface energy which led to energetically favorable (111)-oriented CFO spinel surfaces. This was valid for the CFO thin film deposited at Ts = 100–350 °C. Apart from the ***γ***_***s***_ minimizing effect, ***M***_***hkl***_***ε***^***2***^ also influences the total energy and the film orientation. ***M***_***hkl***_***ε***^***2***^ depends on the crystallographic orientations (***hkl***), and the effective ***M***_***hkl***_ of the grains. The ***M***_***hkl***_ can be calculated as a function of the grain orientation (***hkl***) and the stiffness constants (***C***_***11***_, ***C***_***12***_ and ***C***_***44***_) as ***M***_***hkl***_ = ***C***_***11***_ + ***C***_***12***_ + ***K*** − [***2***(***C***_***12***_ − ***K***)^2^/(***C***_***12***_ + ***2K***)], where K = (***2C***_***44***_ − ***C***_***11***_ + ***C***_***12***_)(***h***^***2***^***k***^***2***^ + ***k***^***2***^***l***^***2***^ + ***h***^***2***^***l***^***2***^)/(***h***^***2***^ + ***k***^***2***^ + ***l***^***2***^)^2^,^20^.

The CFO thin film deposited at Ts = RT, 50 °C and ≥350 °C should have the values of ***M***_***hkl***_***ε***^***2***^, ***M***_***hkl** (**111***)_ (3.68 × 10^12^ dynes/cm^2^) > ***M***_***hkl** (**311***)_ (3.09 × 10^12^ dynes/cm^2^). The smaller strain energy density for (311) leads to a random orientation of the CFO thin film rather than an orientation in the (111) direction because the formation of (111) texture films possesses larger strain energy density than the formation of other textures. The discussion from here onwards is primarily related to the CFO thin films deposited at Ts = RT, post-annealed at 800 °C (hereafter referred to as CFO_RT(*Random*)_) and Ts = 250 °C and post-annealed at 400 °C (hereafter referred to as CFO_250(*111*)_) because these films showed the strongest ‘*random*’ and ‘(*111*)’ orientations, respectively.

The variation of average out-of-plane (OOP) lattice parameter (***a***_***⊥***_; c-axis) with the substrate deposition temperature for all CFO thin films is depicted in Fig. 2(c). It is observed that the ***a***_***⊥***_ lattice parameter increased from 8.344 to 8.396 Å with the increase in the substrate temperature from Ts = RT to 250 °C. However, the further increment in the Ts (>250 °C) again shrank the lattice parameter. It remained almost unchanged for the (111)-oriented CFO thin films deposited in the range of Ts = 100–250 °C, revealing that there was no detective lattice distortion, and it fully relaxed toward the bulk value of CFO (a = b = c = 8.391 Å). This likely also occurred because the CFO thin films deposited in this range were in a thermo-equilibrium state without a significant intrinsic strain, which is important because CFO is known to be highly magnetostrictive. However, the ***a***_***⊥***_lattice parameter for the randomly oriented CFO thin films deposited at Ts = RT, 50 °C and ≥350 °C were significantly lower than the (111)-oriented and CFO bulk values, indicating the contraction in the OOP direction. Therefore, randomly oriented CFO thin films were in an out-of-plane compressive residual strain that was Ts dependent. To study the strain effect, we determined the in-plane lattice parameter (***a***_***||***_; a-axis) by employing the XRD from the in-plane direction of the CFO film (Supplementary Fig. 2). The calculated lattice parameters for the most intense (311) peak for the CFO_RT(*Random*)_ were ***a***_***⊥***_ = 8.342 Å and ***a***_***||***_ = 8.427 Å. The c/a (***a***_***⊥***_/***a***_***||***_) ratio was 0.989, which confirmed the face-centered cubic to face-centered tetragonal ( ***fct***) distortion of randomly oriented CFO.

The in-plane and out-of-plane strains (***ε***) were calculated using the formula: ***ε*** = (***a*** − ***a***_***0***_)/***a***_***0***_, where ***a*** is the in-plane (***a***_***||***_) or out-of-plane (***a***_***⊥***_) lattice parameters and ***a***_***0***_ is the bulk CFO unstressed lattice parameter. CFO_RT(*Random*)_ post-annealed at 800 °C showed the highest out-of-plane compressive strain (***ε***_***⊥***_) of −0.583% and an in-plane tensile strain (***ε***_***||***_) of 0.425%. This marked difference between the ***ε***_***⊥***_ and ***ε***_***||***_ strains was attributed to the fact that the lattice parameters (***a***_***||***_) and (***a***_***⊥***_) differed by 1%. One of the reasons for the observed strain in post-annealed CFO_RT(*Random*)_ film was attributed to the difference in the thermal expansion coefficient (***α***) between the SiO_2_/Si substrate (3.5 × 10^−6^/K) and CFO (1 × 10^−5^/K). This difference in ***α*** caused tension along the in-plane direction during the cooling of the CFO thin film to room temperature after post-annealing. However, the observed strain and therefore oxygen vacancies may also have contributed to the tension, which is discussed later on in the XPS results. By contrast, (111)-oriented CFO thin films deposited at Ts = 100–325 °C showed a small OOP tensile strain, ∼0.06%, which confirmed the lattice relaxation.

**Figure 3:**
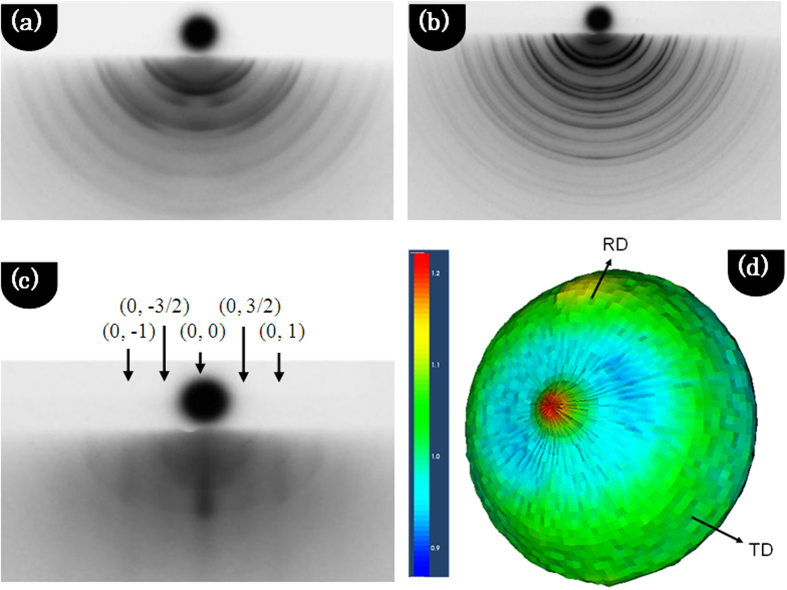
RHEED pattern of the CoFe2O4 thin film along the 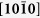 direction. (**a**) CFO_RT(*Random*)_ (post-annealed at 800 °C), (**b**) CFO_375(*Random*)_ (post-annealed at 400 °C), and (**c**) CFO_250(*111*)_ (post-annealed at 400 °C). At the top (*0, 3/2*) *and* (0, 1) *streaks have been labeled for clarity.* (**d**) 3D pole figure pole figure of CFO_250(*111*)_ (post-annealed at 400 °C).

We studied the *ex-situ* RHEED patterns along the 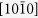 direction in reciprocal space. Figure 3(a,b) shows the RHEED patterns of CFO_RT(*Random*)_ and CFO_375(*Random*)_, respectively, and confirms the excellent crystallinity. In these images, diffraction rings are observed, indicating the random and polycrystalline nature of CFO thin films. Figure 3(c) represents the RHEED pattern of CFO_250(*111*)_; the streaks observed on the RHEED pattern represent the reciprocal lattice. The observed smooth streaks (0, 1) and (0, −1)-type planes corresponded to the FCC sublattice. No spotty pattern was observed that indicated that the deposited film grew in two dimensions. The intermediate (0, 3/2) and (0, −3/2) streaks revealed the characteristic RHEED pattern of the spinel structure. The slight presence of rings around the RHEED streaks suggested the crystalline quality was not excellent, which is related to the post-annealing of the CFO thin film. To obtain more clarity of the (111) orientation in CFO_250(*111*)_, we measured the pole figure of the (111) plane (Fig. 3d). It was observed that a pole was located at the center, which showed that the (111) plane of CFO was parallel to the sample surface and further confirmed that the film had a strong (111) texture.

**Figure 4:**
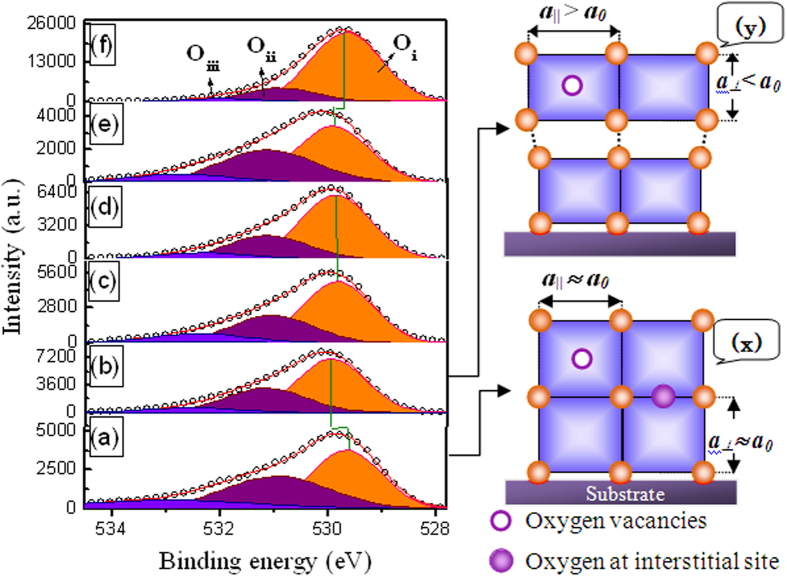
XPS spectra of O1s core level of CFO thin films. (**a**) CFO_RT(*Random*)_ (as deposited), (**b**) CFO_RT(*Random*)_ (post-annealed at 800 °C), (**c**) CFO_250(*111*)_ (as-deposited), (**d**) CFO_250(*111*)_ (post-annealed at 400 °C), (**e**) CFO_375(*Random*)_ (as deposited) and (**f**) CFO_375(*Random*)_ (post-annealed at 400 °C). Schematic (**x**) shows the illustration of the ***fcc*** CFO lattice dimension of the as-deposited CFO_RT(*Random*)_ with oxygen vacancies and oxygen at interstitial sites. (**y**) is the schematic showing the effect of the post-annealing temperature on the CFO_RT(*Random*)_ film. Lattice wall of the bottom layer of CFO thin film is covalently bonded with the substrate and posses the ***fcc*** structure, however as the layer thickness increased it transformed from the ***fcc*** to the ***fct***structure with the removal of oxygen from the interstitial sites.

XPS spectroscopy is the most appropriate method to identify the oxidation state and chemical composition of CoFe_2_O_4_. Figure. 4 shows the XPS spectra of CoFe_2_O_4_ thin films prepared at different synthesis parameters. The XPS spectra primarily involve the Co2p, Fe2p and O1s peaks, and the shape of these peaks depends greatly on the chemical state of the atoms. In the case of Fe, the Fe2p peak was split into two components, Fe2p_1/2_ at 723.5 eV and Fe2p_3/2_ at 710.1 eV, due to spin-orbit coupling (supplementary Fig. 3). It is known that a noticeable satellite peak in the intermediate Fe2p_1/2_ and Fe2p_3/2_ peak appears due to the presence of only Fe^3+^ ions. However, in the present case, the intensity of such a satellite peak (∼718 eV) was less evident. We were more interested in the O1s peak as the CFO film deposition was carried out in Ar gas atmosphere only without introducing the oxygen gas. Oxygen does play a vital role in governing the structural and magnetic properties in an oxygen closed-packed cubic spinel system such as CFO. The O 1s XPS spectra were deconvoluted into three symmetric peaks corresponding to lattice oxide (Fe/Co-O), surface hydroxyl (Fe/Co-OH) and loosely bound oxygen, such as absorbed O^2−^ or adsorbed H_2_O at O_i_ = 529.67–529.94 (±0.05) eV, O_ii_ = 530.89–531.17 (±0.15) eV and O_iii_ = 532.01−532.91 (±0.9) eV, respectively. In the present case, the main peak ‘O_i_’ of O 1 s at 529.91 eV shifted to a lower binding energy at 529.68 eV only in the case of CFO deposited at Ts = 375 °C. This peak almost remained constant at 529.83–529.87 eV for as-deposited and post-annealed CFO_250(*111*)_; therefore, it was considered not to be oxygen deficient. However, more importantly, the O_i_ peak position suggested that the binding energy of the as-deposited CFO_RT(*Random*)_ was 529.67 eV, and after post-annealing, it shifted to a higher binding energy of 529.94 eV.

It should be considered that the amorphous inorganic framework of the CFO thin film deposited at room temperature is porous and may absorb some oxygen from the atmosphere. These absorbed oxygen ions reside at interstitial sites and are diffused out after post-annealing as the film become dense. Because the film is covalently bound to the substrate lattice, the wall density can be increased not in an in-plane direction but in the OOP direction. Because of the increased density, the CFO lattice can distort to relax the strain with oxygen vacancies by compressing the c-axis lattice parameter and expanding the a-axis lattice parameter. We schematically demonstrate this mechanism in Fig. 4 (x and y) for a better understanding. It has been reported that presence of oxygen at interstitial sites may enhance the c-axis lattice parameter of the perovskite structure^21^. As reported earlier that oxygen vacancies increase the c-axis lattice parameter of oxide thin films such as La_0.65_Sr_0.35_MnO_3−δ_/SrTiO_3_^22^, HoMnO_3_/Al_2_O_3_^23^, LaMnO_3_/SrTiO_3_^24^, LaAlO_3_, BiFeO_3_^25^ and (LaSr)CoO/NGO, LSAT^26^. However, our results for post-annealed cubic CFO_RT(*Random*)_ are contrary to these reports, rejecting the possibility of the presence of oxygen at interstitial sites after post-annealing. The shift in O_i_ can be attributed to the increased oxygen vacancies, which decreased the work function and resulted in the O_i_ (O1s) peak shifts towards higher binding energies^27^.

Variations in the O_ii_ and O_iii_ peaks further clarified the oxygen vacancies in the CFO thin film. O_ii_ can also be related to oxygen in oxygen-deficient regions. The position of the O_ii_ peak generally has a higher binding energy by 1.5 eV than O_i_^28^. However, as per Fig. 4 it is higher only by1.25 (±0.03) eV, which may be due to the contribution from oxygen-deficient regions. The calculated O_ii_/O_i_ ratios were approximately 0.49 (±0.1), 0.38 (±0.2) and 0.18 (±0.1) for the post-annealed CFO_RT(*Random*)_, CFO_250(*111*)_ and CFO_375(*Random*)_, respectively. A decrease in the ratio of O_ii_/O_i_ with the increase in the substrate temperature confirmed the reduction of the oxygen vacancies in CFO thin films. It should be noted here that the contribution of OH to the O_ii_ region may not be ignored; this become even more significant for the CFO_250(*111*)_ film because no peak shift of O_i_ was observed. As discussed earlier, the non-evident or very weak Fe^3+^-state intermediate satellite peak between Fe2p_3/2_ and Fe2p_1/2_ at ∼718 eV indicates that Fe^2+^ ions produced by the reduction from Fe^3+^ ions which is due to oxygen deficiency. Furthermore, to sustain the charge neutrality, oxygen vacancies in the film were compensated by a transformation of the Fe cation oxidation state, from Fe^3+^ to Fe^2+^ ions. Therefore, the spinel Fe_3_O_4_ phase may be formed with the reduction of oxygen in the CFO films during sputtering.

Quantitative analysis of overall chemical composition is found to be Co_0.95_Fe_2.05_O_4-δ_, Co_0.98_Fe_2.02_O_4_ and Co_0.99_Fe_2.01_O_4_ for the post-annealed CFO_RT(*Random*)_, CFO_250(*111*)_ and CFO_375 (*Random*)_ thin films, respectively. The cation distribution by the XPS data was estimated as (Co_0.38_Fe_0.62_)^A^[Co_0.57_Fe_1.43_]^B^O_4-δ_ and (Co_0.15_Fe_0.85_)^A^[Co_0.83_Fe_1.17_]^B^O_4_ for post-annealed CFO_RT(*Random*)_ and CFO_250(*111*)_ films, respectively. As reported earlier, the Fe^2+^ ions in CFO occupied only B-sites, thus pushing the Co^2+^ ions to the A-site^29^.

**Figure 5:**
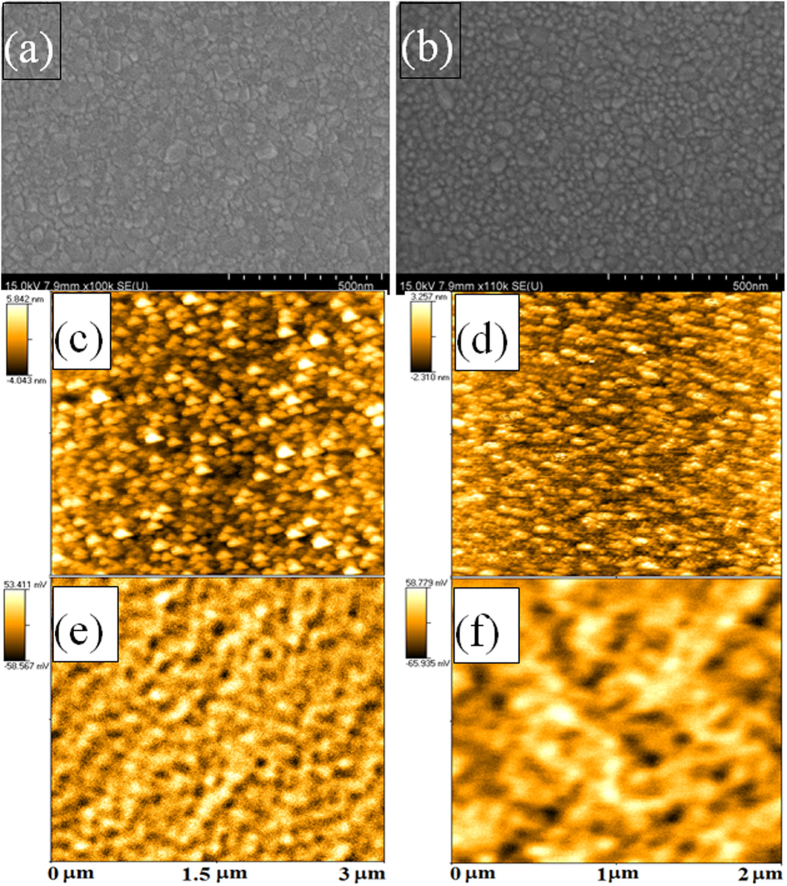
SEM, AFM and MFM images of CFO thin film. (**a**) SEM of CFO_RT(*Random*)_ post-annealed at 800 °C for 4 h; (**b**) SEM of CFO_250(*111*)_ post-annealed at 400 °C for 4 h; (**c**) AFM image and (**d**) its corresponding MFM image of post-annealed CFO_RT(*Random*)_, whereas (**e**) AFM image and (**f**) is its corresponding MFM image of post-annealed CFO_250(*111*)_ thin film.

The surface morphology of CFO thin films was observed by SEM (Fig. 5). The film surface was smooth for CFO_RT(*Random*)_ and CFO_250(*111*)_. The disc-shape grains were of an irregular size and shape, giving rise to the random orientation for the CFO_RT(*Random*)_ (Fig. 5a). In contrast to these uniformly isolated spherical-shaped grains, a narrow grain-size distribution was observed for the CFO_250(*111*)_ (Fig. 5b). As the Ts increased to 375 °C (CFO_375(*Random*)_), some region of the CFO thin film melted, exposing some voids and cracks (Supplementary Fig. 4) gave rise the random orientation for CFO_375(*Random*)_. The average grain size estimated from these images are 35, 30 and 110 nm for Ts = RT, 250 °C and 375 °C respectively. The representative AFM images are depicted in Fig. 5, exhibiting grains with different shapes. Post-annealing CFO_RT(*Random*)_ had a root mean square roughness (RMS) of 1.856 nm, whereas for CFO_250(*111*),_ it was 0.903 nm. The corresponding MFM images show that it consisted of domains with a cluster-like structure for CFO_RT(*Random*)_ film in which magnetization was confined up and down with dark and bright contrasts, respectively.

**Figure 6:**
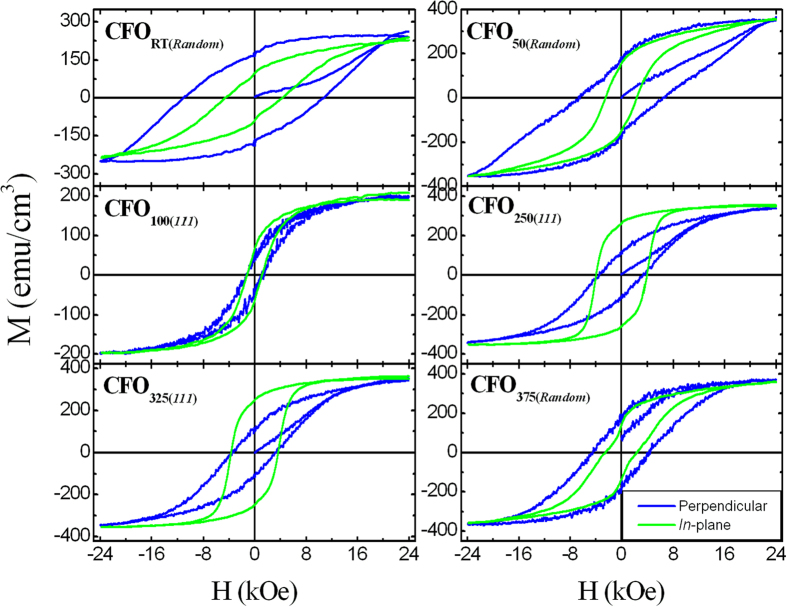
Room temperature variation of magnetization (M) with applied magnetic field (H) of post-annealed CFO thin films. M-H loops of CFO_RT(*Random*)_, CFO_50(*Random*)_ and CFO_375(*Random*)_ are of randomly oriented CFO thin film where perpendicular coercivity is higher than that of in-plane coercivity. In these cases magnetic easy-axis is along perpendicular (c-axis) direction. M-H loops of CFO_100(*111*)_, CFO_250(*111*)_ and CFO_325(*111*)_ are of (111) oriented CFO thin film where in-plane coercivity is higher than that of perpendicular coercivity. In these cases magnetic easy-axis is along in-plane direction (a-axis) direction.

**Figure 7:**
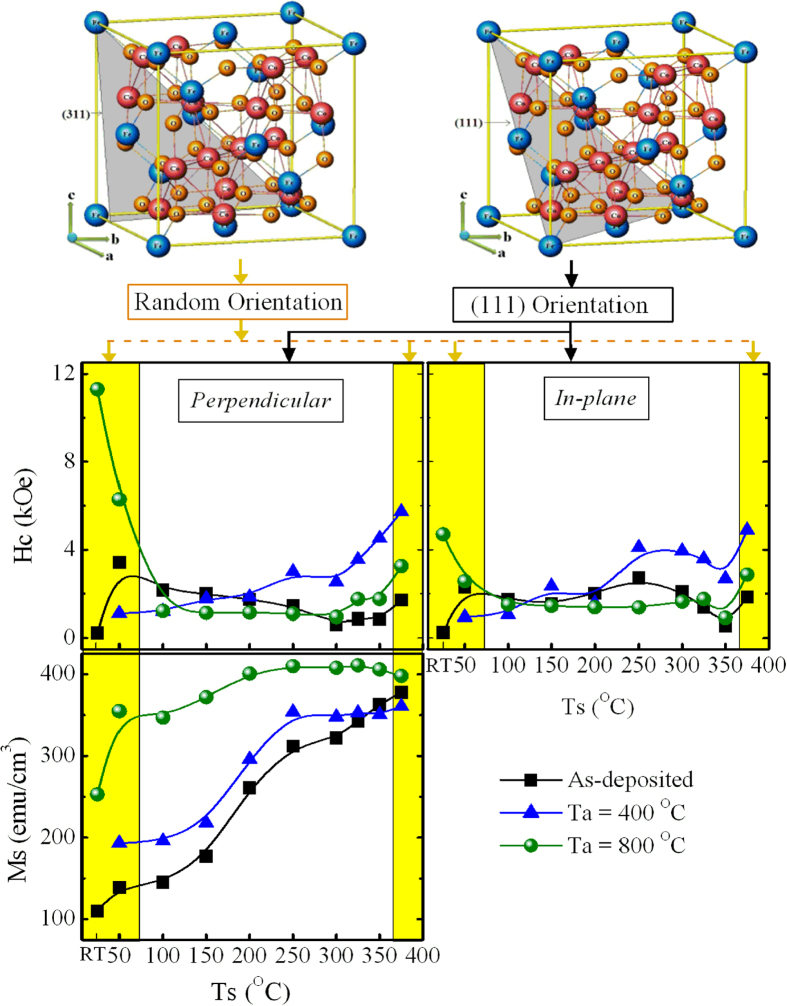
Variation of coercivity (Hc) and saturation magnetization (Ms) with different substrate temperature (Ts) of CoFe_2_O_4_ thin film. The magnetization measurements were carried out in ‘perpendicular’ and ‘In-plane’ directions of the film surface. Yellow pillar region in the Ms, and ‘Hc’ graphs denotes the magnetic measurements of randomly oriented CFO thin film. Ms values are considered to be remain unchanged for both ‘perpendicular’ and ‘In-plane’ directions.

To reveal the co-relation of crystal orientation with magnetization, the magnetic behavior of CFO thin film was analyzed by performing VSM. The hysteresis loops of the representative CFO thin film samples obtained by the VSM are depicted in Fig. 6. Magnetic properties such as saturation magnetization (***Ms***), the remanent ratio (***R*** = ***Mr/Ms***) and coercivity (***Hc***) extracted from hysteresis loops of as-deposited and post-annealed (400 and 800 °C) CFO thin film and are presented in Fig. 7 and supplementary Fig. 5. It is noticed that all the post-annealed randomly oriented CFO films possess a large perpendicular coercivity compared to in-plane coercivity and exhibit a higher ***Ms**, **Mr***, and ***R*** and a magnetic easy axis in the OOP direction. In contrast, all the (111)-oriented CFO films exhibiting a magnetic easy axis in the in-plane direction show higher in-plane coercivity than perpendicular coercivity and have a higher ***Ms**, **Mr***, and ***R*** along the in-plane direction. As discussed earlier, the lattice of post-annealed CFO_RT(*Random*)_ film was under face cubic tetragonal distortion, which suggests that the magnetoelastic energy (***K***_***me***_) may contribute to the control of the magnetic easy axis, where ***K***_***me***_ is directly proportional to (***a***_***||***_ − ***a***_***⊥***_)^30^. Randomly oriented CFO film has ***K***_***me***_ > 0; therefore, ***K***_***me***_ is uniaxial, which directs the easy axis in the OOP direction. It is exactly opposite for (111)-oriented films, where ***K***_***me***_ < 0 and has a negative value, leading to a biaxial in-plane anisotropy, which makes an easy axis along the in-plane direction. The analysis of ***K***_***me***_ also suggests that all the randomly oriented CFO films were under OOP compressive strain; this strain forced the magnetic easy axes to lie parallel to the compression direction, which caused the perpendicular magnetic anisotropy. By contrast, (111)-oriented films showed little OOP tensile strain, which compensated for compression along the in-plane direction and thus resulted an increase in strain anisotropy. Increased strain anisotropy translated into an increased magnetization along the magnetically easy in-plane direction for (111)-oriented films. Moreover, the IP M-H loop of CFO_250(*111*)_ has strong contribution to magnetic anisotropy of the CFO film. It makes strong demagnetization energy and affects significantly in anisotropy constant. This finding clearly suggested that the crystal orientation and compressive strain drove the direction of the magnetic easy axis in the CFO thin film.

The present work was designed to achieve high coercivity. Randomly oriented post-annealed CFO_RT(*Random*)_ film showed the highest perpendicular coercivity (***Hc***_***⊥***_), 11.27 kOe, with an in-plane coercivity (***Hc***_***||***_) of 4.7 kOe. By contrast, (111)-oriented post-annealed CFO_250(*111*)_ film showed an ***Hc***_***||***_of 4.1 kOe, which was higher than its ***Hc***_***⊥***_ (3.0 kOe). Considering the deposition of CFO on the SiO_2_/Si substrate, it is worth emphasizing that the observed high perpendicular coercivity measured at room temperature in post-annealed CFO_RT(*Random*)_ film was, to the best of our knowledge, greater than 5.37 kOe (91%), compared to the highest coercivity of 5.9 kOe for CFO thin film previously reported by Raghunathan *et al*.^31^. A comparable coercivity in CFO was reported by Yin *et al*.^32^; however, they used single-crystal quartz and (0001) sapphire substrate and employed a high substrate temperature of 550 °C.

The observed very high coercivity in post-annealed CFO_RT(*Random*)_ film can be interpreted in terms of the total uniaxial anisotropy energy (***Ea***) that controls the spin alignment in thin films. ***Ea*** is the sum of several factors, including thickness (***t***), surface (***K***_***s***_), shape (***K***_***sh***_), stress (***K***_***a***_) and magnetocrystalline (***K***_***u***_) anisotropies; therefore, it can be written as ***Ea*** = ***K***_***s***_/***t*** + ***K***_***sh***_ + ***K***_***a***_ + ***K***_***u***_, where ***K***_***s***_, ***K***_***sh***_, and ***K***_***a***_ and ***K***_***u***_ are the extrinsic and intrinsic contributions, respectively. A positive ***Ea*** indicates an easy axis is in the perpendicular direction and OOP magnetic anisotropy, whereas a negative value of ***Ea*** indicates in-plane direction and in-plane magnetic anisotropy^30^. All CFO thin films under investigation are 40-nm thick; therefore, ***K***_***s***_***/t*** can be neglected because it is effective for much thinner film. ***K***_***sh***_ is equivalent to −***2πMs***^***2***^; the calculated values are −0.4 × 10^6^ erg/cm^3^ and −1.05 × 10^6^ erg/cm^3^ for CFO_RT(*Random*)_ and CFO_250(*111*)_ films, respectively. Because the ***K***_***sh***_ is negative, it contributes only to in-plane magnetization^33^. Relating ***K***_***sh***_ with **K**_**me**_, **K**_**me**_ has a positive value for CFO_RT(*Random*)_, whereas it is negative for the CFO_250(*111*)_ film. This indicates that a positive **K**_**me**_ is sufficient to overcome a negative ***K***_***sh***_ in CFO_RT(*Random*)_; therefore, ***K***_***sh***_ has less of an effect and can be neglected. CFO is known to have a high ***K***_***u***_ because of the spin orbit stabilized ground state and unquenched orbital momentum, caused by a trigonal crystal field on the Co^2+^ cations. However, according to the single-ion model, this is true only when CFO possesses a fully inverse spinel structure, where Co^2+^ ions occupy only the octahedral site. As observed from the XPS results, Co^2+^ ions occupy a tetrahedral A site in a greater amount, suggesting that the CFO_RT(*Random*)_ film possesses a partial inverse spinel structure. Therefore, ***K***_***u***_ does not play a significant role in the observed high ***Hc***_***⊥***_ in the CFO_RT(*Random*)_ film.

The stress (***σ***) was calculated using the relation ***σ*** = ***Yε***, where ***Y*** is the Young’s modulus for CFO (Y = 1.5 × 10^12^ dyne/cm^2^)^34^. The in-plane (***σ***_***||***_) and out-of-plane (***σ***_***⊥***_) stresses calculated for the CFO_RT(*Random*)_ film were found to be 6.37 × 10^9^ and −8.74 × 10^9^ dyne/cm^2^, respectively, whereas it was ***σ***_***⊥***_ = 0.89 × 10^9^ dyne/cm^2^ for the CFO_250(*111*)_ film. Here, negative sign indicates the compression whereas positive sign indicates the tension. Using these stress values, the stress-induced anisotropy constant (***K***_***a***_) was estimated, ***K***_***a***_ = (3/2)***λ σ***, where ***λ*** is the magnetostriction coefficient and is taken as; ***λ***_***100***_ = −590 × 10^−6^ 34,35,36. The estimated value of ***K***_***a***_ for post-annealed CFO_RT(*Random*)_ is 7.7 × 10^6^ erg/cm^3^ in perpendicular direction which is higher than bulk the CFO (2 × 10^6^ ergs/cm^3^). Therefore the total uniaxial anisotropy energy (***Ea***) in post-annealed CFO_RT(*Random*)_ film is mainly governed by the ***K***_***a***_ and thus played a deciding role in the high ***Hc***_***⊥***_ for CFO_RT(*Random*)_ film. By contrast, the moderate ***Hc*** observed in the CFO_250(*111*)_ film correlated with a lower ***K***_***a***_ as a result of lattice-strain relaxation. The Ms value for the CFO_RT(*Random*)_ film was slightly lower than that of bulk CFO and CFO_250(*111*)_. This may be attributed to strain, a partially inverse spinel structure, and/or antiphase boundaries, which form when islands that nucleate at different areas of the substrate merge and are out of phase with each other^37^. The lower ***Ms*** for CFO_RT(*Random*)_ is also attributed to the oxygen deficiency, which led to a decrease in the super-exchange interaction of the Co^2+^ cation and the O^2−^ anion.

## Conclusions

40-nm CFO thin film with a (111) orientation and a (311)-random orientation was prepared by DC magnetron sputtering on an SiO_2_/Si substrate. CFO was deposited at different substrate temperatures, ranging from room temperature to 375 °C. The (111) orientation was achieved at a very low substrate temperature, 100 °C; however, this orientation was strongest at a substrate temperature of 250 °C, as evidenced by the XRD patterns and rocking-curve measurements and the RHEED images. In the case of randomly oriented CFO, the out-of-plane lattice constant was much smaller than that of the in-plane lattice constant, revealing the fact that randomly oriented CFO was under compressive residual strain, possessing an ***fct*** structure. By contrast, (111)-oriented CFO showed a relaxed crystal structure and was under slight tensile strain with an average grain size of 30 nm. The XPS results showed that randomly oriented post-annealed CFO had some oxygen deficiency; this deficiency and the increase in film density produced compressive strain in this film. It was observed that randomly oriented CFO showed a magnetic easy axis along the perpendicular direction (c-axis); exactly the opposite, the (111)-oriented film showed the magnetic easy axis along the in-plane direction (a-axis). Randomly oriented CFO thin film possessed a large perpendicular coercivity of 11.3 kOe. This large coercivity is associated with the compressive strain anisotropy induced by the oxygen deficiencies. The ultra-smooth surface observed from the AFM and the large perpendicular coercivity makes randomly oriented CFO thin film suitable for magnetic recording media. Importantly, the demonstrated novel approach provides a facile way to control and manipulate the magnetic easy axis in CFO thin film on virtually any substrate.

## Additional Information

**How to cite this article**: Shirsath, S. E. *et al*. Switching of magnetic easy-axis using crystal orientation for large perpendicular coercivity in CoFe_2_O_4_ thin film. *Sci. Rep.* **6**, 30074; doi: 10.1038/srep30074 (2016).

## Supplementary Material

Supplementary Information

## Figures and Tables

**Figure 1:**
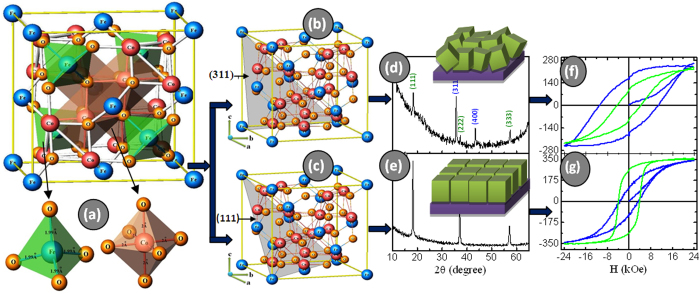
Schematic representation of crystal structure orientation and its effect on magnetic properties. (**a**) Cubic spinel crystal structure of CFO, indicating tetrahedral and octahedral sites. (**b**) A (311)-plane that dominates the random orientation. (**c**) A (111)-plane that dominates (111) orientation in the cubic spinel CFO. (**d**) The XRD pattern of randomly oriented CFO thin film; the inset shows the film texture of polycrystalline film with a random orientation. (**e**) The XRD pattern of (111)-oriented CFO thin film; the inset shows the film texture of epitaxial film with a strong (111) orientation. (**f**) The hysteresis loops of randomly oriented CFO, and (**g**) the hysteresis loops of (111)-oriented CFO, where the blue loop shows the magnetization measured in the perpendicular direction, and the green loop shows the magnetization measured along the in-plane direction.
