# Supplementary material for: Switching of magnetic easy-axis using crystal orientation for large perpendicular coercivity in CoFe2O4 thin film
Source: Sci Rep. 2016 Jul 20;6:30074. doi: 10.1038/srep30074 (PMC4951806; doi:10.1038/srep30074)
Supplement: Supplementary Information [file srep30074-s1.pdf]

**Supplementary Material**

# Switching of magnetic easy-axis using crystal orientation for large perpendicular coercivity in $CoFe_2O_4$ thin film

Sagar E. Shirsath[1,*], Xiaoxi Liu[1], Yukiko Yasukawa[1], Sean Li[2], Akimitsu Morisako[1]

[1] *Spin Device Technology Center, Faculty of Engineering, Shinshu University, Nagano 380-8553, Japan*

[2] *School of Materials Science and Engineering, University of New South Wales, Sydney, NSW 2502, Australia*

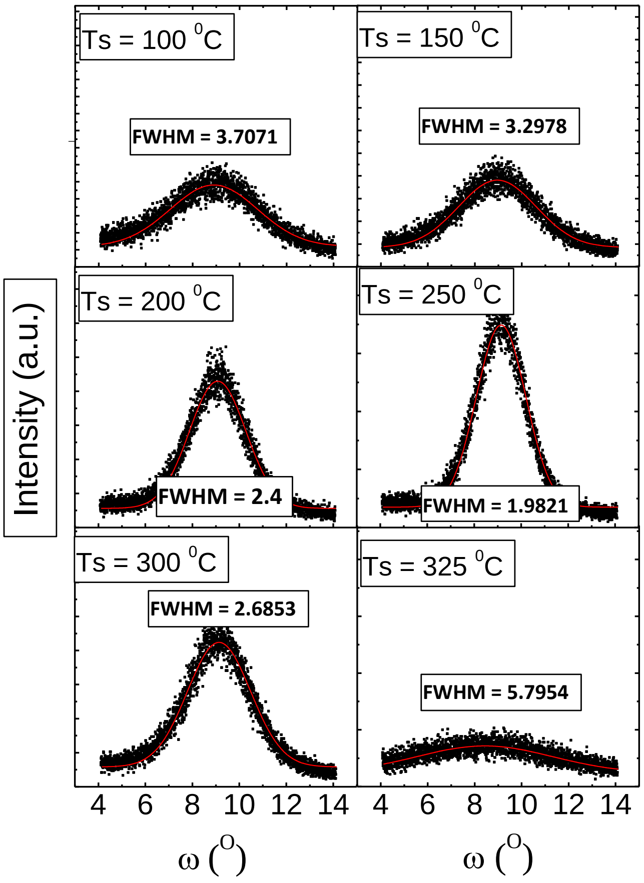


***Supplementary Figure 1:*** *The rocking-curve measurements for the (111) plane of the CFO thin film deposited at different substrate temperatures.*

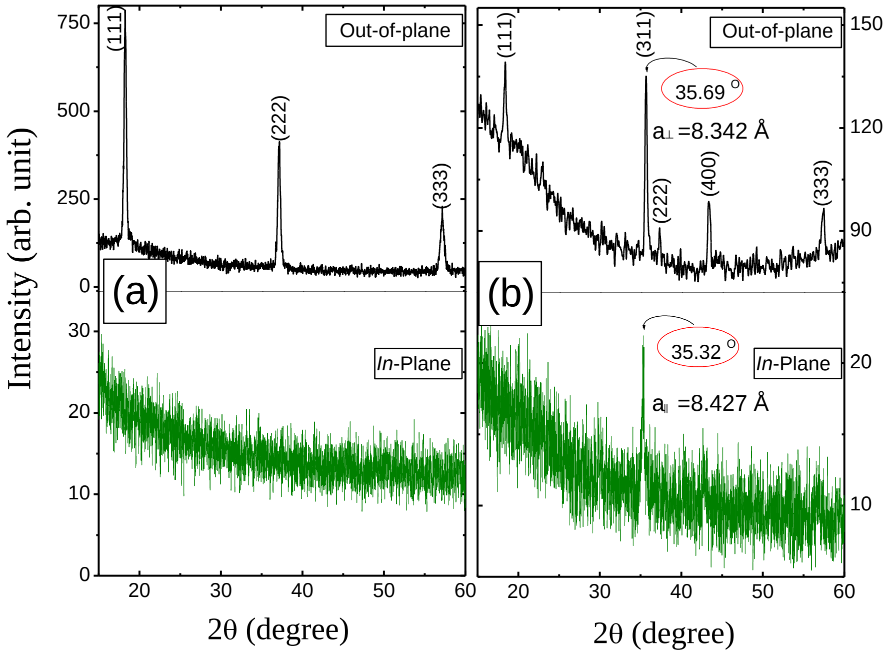


***Supplementary Figure 2: Out-of-plane and in-plane X-ray diffraction patterns of post-annealed CFO thin film****. (**a**) $CFO_{250(111)}$ and (**b**) $CFO_{RT(Random)}$. The value in the red oval shows the 2$\theta$ position of the (311) peak.*

As revealed in Fig. 2(a), even at a very slow scanning rate, no trace of any lattice planes was detected for the (111)-oriented CFO thin film in the in-plane direction, confirming the strong (111) orientation along the c-axis.

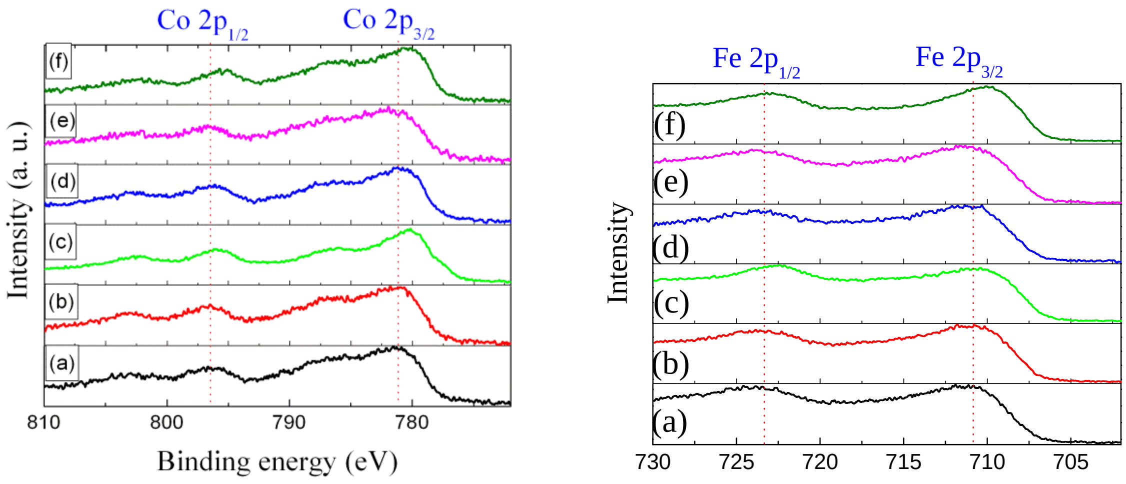


***Supplementary Figure 3: XPS spectra of Co 2p and* Fe 2p *of CFO thin films*. *(a)*** *$CFO_{RT(Random)}$ (as deposited),* ***(b)*** *$CFO_{RT(Random)}$ (post-annealed at 800 ℃),* ***(c)*** *$CFO_{250(111)}$ (as-deposited),* ***(d)*** *$CFO_{250(111)}$ (post-annealed at 400 ℃),* ***(e)*** *$CFO_{375(Random)}$ (as deposited) and* ***(f)*** *$CFO_{375(Random)}$ (post-annealed at 400 ℃).*

The peak at 769 eV is caused by $Co2p_{1/2}$ state accompanying a shake-up satellite peak at 802 eV, while peak at 780 eV with a satellite peak of 786 eV was caused by the characteristics $Co2p_{3/2}$ state. The presence of $Co2p_{1/2}$ and $Co2p_{3/2}$ peaks and the highly intense satellites near them confirmed that the cobalt ions were in a high-spin $Co^{2+}$ oxidation state.

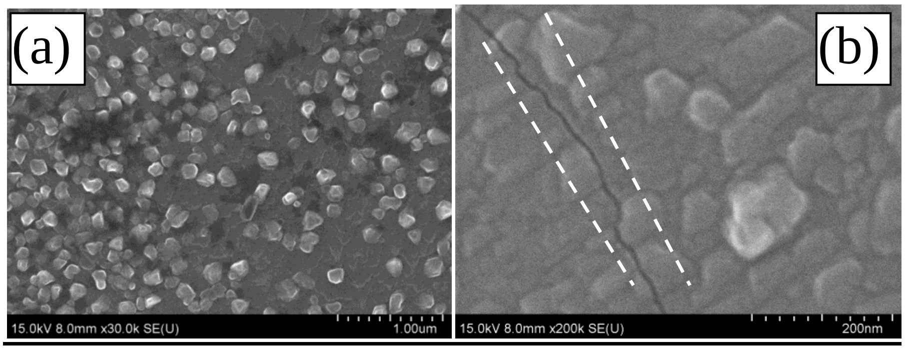


***Supplementary Fig. 4:*** ***(a)*** *SEM of $CFO_{375(Random)}$ post-annealed at 400 ℃ for 4 h.* ***(b)*** *is the magnified SEM image of the same sample where doted area in the inset of image 'b' shows the crack in the film.*

As the Ts increased to 375 °C ($CFO_{375(Random)}$), some region of the CFO thin film melted, exposing some voids and cracks (***Supplementary Fig. 4***) gave rise the random orientation for $CFO_{375(Random)}$. Compared to bulk solid counterparts, the thin film exhibited different thermodynamic characteristics and a lower thermal stability against the phase transformation. Therefore, the melting point of thin film was lower than the equilibrium melting point of bulk material, and it decreased with a decrease in film thickness, particularly in the nanometer thickness[1]. The melting of $CFO_{375(Random)}$ thin film provided useful fingerprints for determining the crystal orientation in this film. The observed film surface, with voids, a melted region and cracks, gave rise the random orientation for $CFO_{375(Random)}$.

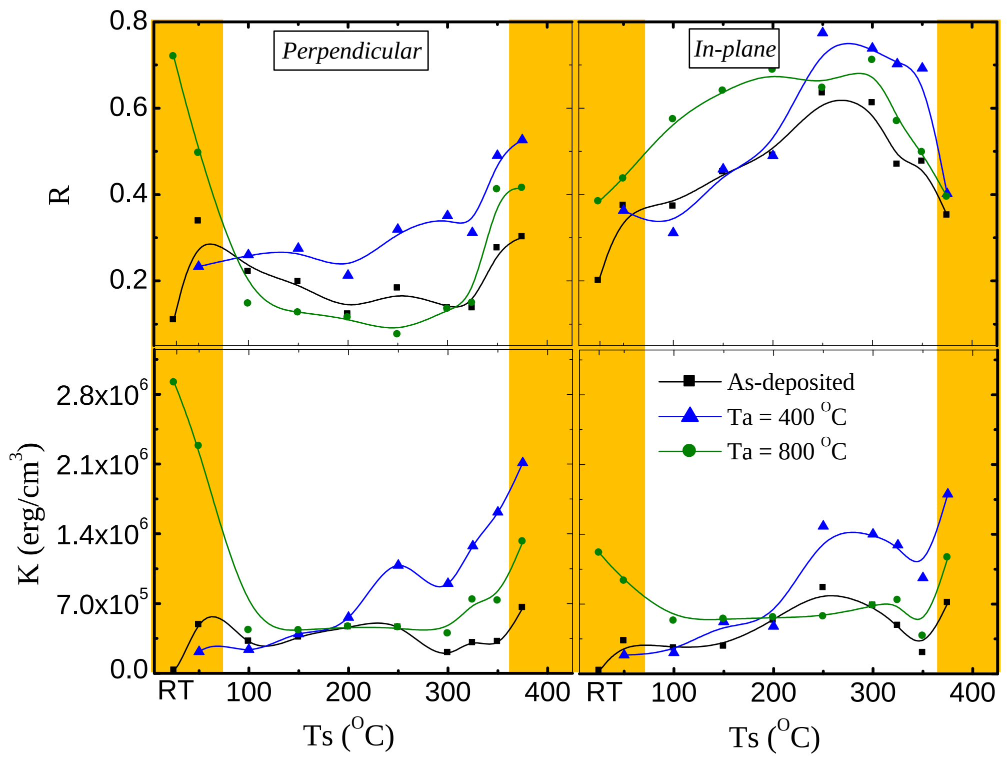


***Supplementary Figure 5: Variation of remanance ratio (R) and anisotropy constant (K) with different substrate temperatures (Ts) of $CoFe_2O_4$ thin films****. Data presented in figure is for the CFO samples those are as-deposited and post annealed at 400 and 800 ℃. The magnetization measurements were carried out in 'perpendicular' and 'In-plane' directions of the film surface. Yellow pillar region in the graphs denotes the magnetic measurements of randomly oriented CFO thin film.*

The cubic anisotropy constant (***K***) was obtained through the Stoner–Wohlfarth theory:[2] $K = (Ms \times Hc)/0.98$ and its variation with substrate temperature is shown in ***Supplementary Fig. 5***. It is observed that the (111)-oriented CFO deposited at Ts = 250 °C posses highest K (1.48

$\times 10^6$ erg/cm$^3$) along in-plane direction which is higher as compare to its perpendicular counterpart. On the other hand randomly oriented CFO deposited at room temperature (Ts = RT) posses highest K (2.91 $\times 10^6$ erg/cm$^3$) along perpendicular direction which is higher as compare to its in-plane counterpart.

References


1. Couchman, P. R. & Jesser, W. A. Thermodynamic theory of size dependence of melting temperature in metals, *Nature* **269**, 481-483 (1977).
2. Stoner E. C. & Wohlfarth, E. P. A mechanism of magnetic hysteresis in heterogeneous alloys. *Philos. Trans. R. Soc., A,* **240**, 599 (1948); Reprinted by *IEEE Trans. Magn*. **27**, 3475 (1991).
